# Supplementary material for: Rhomboid intramembrane protease YqgP licenses bacterial membrane protein quality control as adaptor of FtsH AAA protease
Source: EMBO J. 2020 Jan 13;39(10):e102935. doi: 10.15252/embj.2019102935 (PMC7231995; doi:10.15252/embj.2019102935)
Supplement: Supplementary file 1 — Expanded View Figures PDF [file EMBJ-39-e102935-s001.pdf]

Expanded View Figures

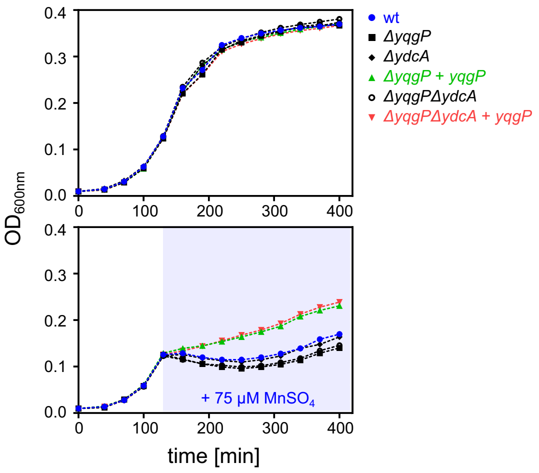

Figure EV1. Analysis of the role of *ydcA* in the phenotypic behaviour of *Bacillus subtilis* during Mn stress.

Growth curves of wild-type (BTM843, Table EV2), *yqgP*-deficient (BTM844, Table EV2), *ydcA*-deficient (BTM1001, Table EV2), *yqgP ydcA*-deficient (BTM1003, Table EV2) and YqgP rescue (BTM845 and BTM1005, Table EV2) strains of *B. subtilis* in M9 minimal medium with limiting magnesium (0.01 mM $MgSO_4$), exposed to manganese stress elicited by adding 75 µM $MnSO_4$ in mid-exponential phase (stress phase denoted by blueish background, in bottom panel). All strains further contain a deletion in the putative manganese efflux pump MntP (*ΔywlD*, Table EV2). Bottom panel shows that manganese is more toxic in both *yqgP*-deficient strains (black squares or open black circles) than in the wild type or *ydcA* rhomboid-deficient strains. The overexpression of YqgP in both *ΔyqgP* and *ΔyqgPΔydcA* strains rescues fitness during manganese stress to above wild-type level. Top panel shows no difference between the strains in the absence of manganese stress.

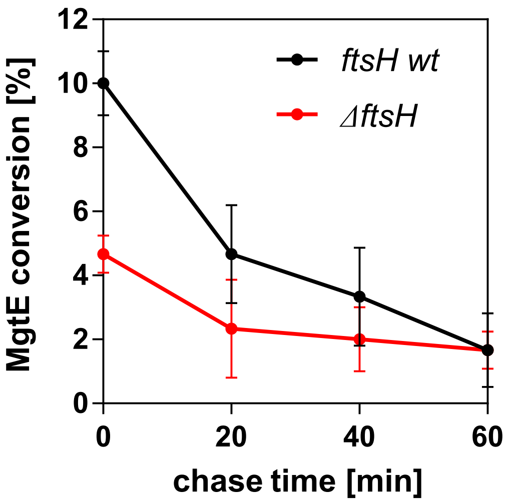

Figure EV2. Stability of MgtE cleavage products depending on the presence of FtsH.

Translational stop chase experiment described in Fig 7 (lanes 13–16 and lanes 37–40) was performed in four independent replicates and quantified by near-infrared Western blotting using α-MgtE antibody as described in Materials and Methods. The conversion of MgtE was plotted against time as average values ± SD, indicating that in the absence of endogenous FtsH, MgtE cleavage products are more stable.

 © 2020 The Authors
