# Supplementary material for: Rhomboid intramembrane protease YqgP licenses bacterial membrane protein quality control as adaptor of FtsH AAA protease
Source: EMBO J. 2020 Jan 13;39(10):e102935. doi: 10.15252/embj.2019102935 (PMC7231995; doi:10.15252/embj.2019102935)
Supplement: Supplementary file 2 — Table EV1 [file EMBJ-39-e102935-s002.docx]

## Table EV1: List of DNA constructs used in this work.

Plasmid constructs used in this work and referred to in the main text are listed below.

| **Plasmid name** | **Description** | **Source** |
| --- | --- | --- |
| **For *B. subtilis*** | | |
| pTM13 | *amyE::Phyperspank-yqgP (spec)* | This work |
| pTM26 | *amyE::Phyperspank-yqgP-sfGFP (spec)* | This work |
| pTM31 | *amyE::Phyperspank-yqgP S288A(spec)* | This work |
| pTM79 | *ycgO::PmgtE-FLAG-mgtE (kan)* | This work |
| pTM89 | *ycgO::Phyperspank-FLAG-mgtE (erm)* | This work |
| pTM92 | *amyE::Pxyl (spec)* | This work |
| pTM93 | *amyE::Pxyl-yqgP (spec)* | This work |
| pTM94 | *amyE::Pxyl-yqgP S288A (spec)* | This work |
| pPR155 | *amyE::Pxyl-yqgP Δ1-178 (spec)* | This work |
| pPR157 | *amyE::Pxyl-yqgP Δ388-507 (spec)* | This work |
| pTM89 | *ycgO::Phyperspank-FLAG-mgtE (erm)* | This work |
| pPR290 | *xkdE::Pxyl-MBP-FLAG-pstatA I5G,I10G-Trx-HA (erm, lin)* | This work |
| pJB216 | *amyE::Pxyl-yqgP D29A (spec)* | This work |
| pJB217 | *amyE::Pxyl-yqgP D37A (spec)* | This work |
| pJB218 | *amyE::Pxyl-yqgP H49A (spec)* | This work |
| pJB219 | *amyE::Pxyl-yqgP D50A (spec)* | This work |
| pJB221 | *amyE::Pxyl-yqgP D52A (spec)* | This work |
| pJB222 | *amyE::Pxyl-yqgP D60A (spec)* | This work |
| pJB223 | *amyE::Pxyl-yqgP E90A (spec)* | This work |
| pWX467 | *loxP-erm-loxP casette* | Gift from D.Z. Rudner |
| pGP886 | *xkdE::Pxyl-YFP-NTD (erm, lin)* | Gift from L. Krásný |
| **For *E. coli*** |  |  |
| pJB171 | *yqgP 1-177-TEV-6×His in pET25b* | This work |
| pJB179 | *6×His-TEV-yqgP 384-507 in pHIS2* | This work |
| pJB184 | *GST-6×His-TEV-mgtE 2-275 in pGEX6P1* | This work |
| pBM9 | *6×His-yqgP 1-170 in pRSET-A (ThermoFisher)* | This work |
| pBM11 | *6×His-yqgP 385-507 in pRSET-A* | This work |
