# Supplementary material for: Rhomboid intramembrane protease YqgP licenses bacterial membrane protein quality control as adaptor of FtsH AAA protease
Source: EMBO J. 2020 Jan 13;39(10):e102935. doi: 10.15252/embj.2019102935 (PMC7231995; doi:10.15252/embj.2019102935)
Supplement: Supplementary file 3 — Table EV2 [file EMBJ-39-e102935-s003.docx]

## Table EV2: List of *Bacillus subtilis* strains used in this work

*Bacillus subtilis* strains that were used in this work and referred to in the main text are listed below. BGSC, Bacillus Genetic Stock Center at [www.bgsc.org](http://www.bgsc.org); NBRP, National BioResource Project at www.nbrp.jp;

| **Strain ID** | **Genotype** | **source** |
| --- | --- | --- |
| BTM2 | *Wild type 168 trpC+* | BGSC, ([Nicolas et al., 2012](#_ENREF_51)) |
| BTM70 | *168 trpC+ amyE::Phyperspank-yqgP-sfGFP (spec)* | This work |
| BTM78 | *168 trpC+ ΔyqgP::cat* | This work |
| BTM84 | *168 trpC+ ΔyqgP::cat,* *amyE::Phyperspank*-*yqgP-sfGFP (spec)* | This work |
| BBM1 | *168 trpC+ ΔyqgP::cat,* *amyE::Phyperspank*-*yqgP.S288A-sfGFP (spec)* | This work |
| BTM462 | *168 trpC+ ftsH::erm* | BGSC, ([Koo, Kritikos et al., 2017](#_ENREF_38)) |
| BTM501 | *168 trpC+ ΔyqgP::cat amyE::Phyp-yqgP (spec)* | This work |
| BTM502 | *168 trpC+ ΔyqgP::cat amyE::Phyp-yqgP S288A (spec)* | This work |
| BTM610 | *168 trpC+ ΔyqgP::cat (spec) ycgO::Phyp-FLAG-mgtE (erm)* | This work |
| BTM611 | *168 trpC+ ΔyqgP::cat amyE::Phyp-yqgP (spec) ycgO::Phyp-FLAG-mgtE (erm)* | This work |
| BTM612 | *168 trpC+ ΔyqgP::cat amyE::Phyp-yqgP S288A (spec) ycgO::Phyp-FLAG-mgtE (erm)* | This work |
| BTM659 | *168 trpC+ ΔyqgP::erm* |  |
| BTM677 | *168 trpC+ ΔyqgP* | This work |
| BTM799 | *168 trpC+ ΔyqgP ftsH::erm* | This work |
| BTM795 | *168 trpC+ ΔyqgP::cat ftsH::erm* | This work |
| BTM796 | *168 trpC+ ΔyqgP::cat amyE::Phyperspank-yqgP (spec) ftsH::erm* | This work |
| BTM797 | *168 trpC+ ΔyqgP::cat amyE::Phyperspank-yqgP S288A (spec) ftsH::erm* | This work |
| BTM843 | *168 trpC+ ywlD::erm* | This work |
| BTM844 | *168 trpC+ ΔywlD::erm ΔyqgP::cat* | This work |
| BTM845 | *168 trpC+ ΔywlD::erm ΔyqgP::cat amyE::Phyperspank-yqgP (spec)* | This work |
| BTM872 | *168 trpC+ ΔyqgP amyE::Pxyl-yqgP Δ1-178 (spec) ftsH::erm* | This work |
| BTM873 | *168 trpC+ ΔyqgP amyE::Pxyl-yqgP Δ388-507 (spec) ftsH::erm* | This work |
| BTM874 | *168 trpC+ ΔyqgP amyE::Pxyl-yqgP (spec) ftsH::erm* | This work |
| BTM875 | *168 trpC+ ΔyqgP amyE::Pxyl-yqgP S288A (spec) ftsH::erm* | This work |
| BTM886 | *168 trpC+ ΔyqgP amyE::Pxyl-yqgP Δ1-178, S288A (spec) ftsH::erm* | This work |
| BTM888 | *168 trpC+ ΔyqgP amyE::Pxyl-yqgP Δ388-507, S288A (spec) ftsH::erm* | This work |
| BTM1001  BTM1003  BTM1005  BS50 | 168 trpC+ *ΔywlD::erm ydcA::kan*  168 trpC+ *ΔywlD::erm ydcA::kan yqgP::cat*  168 trpC+ *ΔywlD::erm ydcA::kan yqgP::cat amyE::Phyperspank-yqgP (spec)*  *168 trpC+ ΔyqgP::cat amyE::Phyp-yqgP (spec) ΔlysA::erm* | This work  This work  This work  This work |
| BS51 | *168 trpC+ ΔyqgP::cat amyE::Phyp-yqgP S288A (spec) ΔlysA::erm* | This work |
| BS72 | *168 trpC+ ΔyqgP amyE::Pxyl-yqgP (spec)* | This work |
| BS73 | *168 trpC+ ΔyqgP amyE::Pxyl-yqgP S288A (spec)* | This work |
| BS55 | *168 trpC+ ΔyqgP amyE::Pxyl-yqgP Δ1-178 (spec)* | This work |
| BS57 | *168 trpC+ ΔyqgP amyE::Pxyl-yqgP Δ388-507 (spec)* | This work |
| BS184 | *168 trpC+ ΔyqgP xkdE::Pxyl-MBP-FLAG-TatA I5G,I10G-Trx-HA (erm, lin)* | This work |
| BS185 | *168 trpC+ ΔyqgP amyE::Pxyl-yqgP d1-178 (spec) xkdE::Pxyl-MBP-FLAG-psTatA I5G,I10G-Trx-HA (erm, lin, lin)* | This work |
| BS186 | *168 trpC+ ΔyqgP amyE::Pxyl-yqgP d388-507 (spec) xkdE::Pxyl-MBP-FLAG-psTatA I5G,I10G-Trx-HA (erm, lin)* | This work |
| BS187 | *168 trpC+ ΔyqgP amyE::Pxyl-yqgP (spec) xkdE::Pxyl-MBP-FLAG-psTatA I5G,I10G-Trx-HA (erm, lin)* | This work |
| BS196 | *168 trpC+ ΔyqgP amyE::Pxyl-yqgP D29A (spec) xkdE::Pxyl-MBP-FLAG-psTatA I5G,I10G-Trx-HA (erm, lin)* | This work |
| BS197 | *168 trpC+ ΔyqgP amyE::Pxyl-yqgP D37A (spec) xkdE::Pxyl-MBP-FLAG-psTatA I5G,I10G-Trx-HA (erm, lin)* | This work |
| BS198 | *168 trpC+ ΔyqgP amyE::Pxyl-yqgP H49A (spec) xkdE::Pxyl-MBP-FLAG-psTatA I5G,I10G-Trx-HA (erm, lin)* | This work |
| BS199 | *168 trpC+ ΔyqgP amyE::Pxyl-yqgP D50A (spec) xkdE::Pxyl-MBP-FLAG-psTatA I5G,I10G-Trx-HA (erm, lin)* | This work |
| BS201 | *168 trpC+ ΔyqgP amyE::Pxyl-yqgP D52A (spec) xkdE::Pxyl-MBP-FLAG-psTatA I5G,I10G-Trx-HA (erm, lin)* | This work |
| BS202 | *168 trpC+ ΔyqgP amyE::Pxyl-yqgP D60A (spec) xkdE::Pxyl-MBP-FLAG-psTatA I5G,I10G-Trx-HA (erm, lin)* | This work |
| BS203 | *168 trpC+ ΔyqgP amyE::Pxyl-yqgP E90A (spec) xkdE::Pxyl-MBP-FLAG-psTatA I5G,I10G-Trx-HA (erm, lin)* | This work |
| BKE23380 | *168 trpC2 ΔlysA::erm* | BGSC |
