# Supplementary material for: Rhomboid intramembrane protease YqgP licenses bacterial membrane protein quality control as adaptor of FtsH AAA protease
Source: EMBO J. 2020 Jan 13;39(10):e102935. doi: 10.15252/embj.2019102935 (PMC7231995; doi:10.15252/embj.2019102935)
Supplement: Supplementary file 4 — Table EV3 [file EMBJ-39-e102935-s004.docx]

## Table EV3: NMR restraints and structural statistics for the final water-refined set of YqgPNTD domain structures.

Parameters were generated by the Protein Structure Validation Software suite, as specified in the Methods section.

|  |  |
| --- | --- |
| Non-redundant distance and angle constrains |  |
| Total number of NOE restraints | 2517 |
| Short-range NOEs |  |
| Intra-residue (i = j) | 290 |
| Sequential (\| i - j \| = 1) | 690 |
| Medium-range NOEs (1 < \| i - j \| < 5) | 542 |
| Long-range NOEs (\| i - j \| ≥ 5) | 995 |
| Torsion angles | 300 |
| Hydrogen bond constraints | - |
| Total number of restricting constraints | 2817 |
| Total number of restricting constraints per restrained residue | 5.4 |
| Residual constraint violations |  |
| Distance violations per structure |  |
| 0.1 – 0.2 Å | 3.63 |
| 0.2 – 0.5 Å | 1.63 |
| > 0.5 Å | 0 |
| r.m.s. of distance violation per constraint | 0.01 Å |
| Maximum distance violation | 0.49 Å |
| Dihedral angle viol. per structure |  |
| 1 – 10 ° | 2.93 |
| > 10 ° | 0 |
| r.m.s. of dihedral violations per constraint | 0.33° |
| Maximum dihedral angle viol. | 5.0° |
| Ramachandran plot summary |  |
| Most favoured regions | 94.6 % |
| Additionally allowed regions | 4.9 % |
| Generously allowed regions | 0.4 % |
| Disallowed regions | 0.0 % |
| r.m.s.d. to the mean structure | *all/ordered^1^* |
| All backbone atoms | 3.0/1.0 Å |
| All heavy atoms | 3.5/1.5 Å |
| ***PDB entry*** | **6R0J** |
| ***BMRB accession code*** | **34376** |
