# Supplementary material for: Rhomboid intramembrane protease YqgP licenses bacterial membrane protein quality control as adaptor of FtsH AAA protease
Source: EMBO J. 2020 Jan 13;39(10):e102935. doi: 10.15252/embj.2019102935 (PMC7231995; doi:10.15252/embj.2019102935)
Supplement: Supplementary file 5 — Table EV4 [file EMBJ-39-e102935-s005.docx]

## Table EV4: List of proteins structurally similar to YqgP_NTD_.

The top 20 PDB matches from a Dali server search are displayed and sorted by the Z-score, which indicates a significant similarity match when higher than 2. The quality of the alignment is further measured by RMSD (Root-Mean-Square deviation), L_ali_ (alignment length, or number of structurally equivalent residues), nres (number of aligned protein residues), and %id (sequence identity).

| **No** | **Name** | **Z-score** | **RMSD** | **L_ali_** | **nres** | **%id** | **PDB entry** |
| --- | --- | --- | --- | --- | --- | --- | --- |
| 1 | Nt.BspD6I nicking endonuclease from *Bacillus* | 6.8 | 4.2 | 122 | 594 | 11 | 4wl5 |
| 2 | DNA-directed RNA polymerase II- largest subunit from *Saccharomyces cerevisiae* | 6.5 | 3.5 | 111 | 215 | 8 | 1i3q |
| 3 | Human DNA-directed RNA polymerase II- subunit RPB1 | 6.1 | 3.5 | 107 | 210 | 9 | 5iy6 |
| 4 | Restriction endonuclease FokI from *Planomicrobium okeanokoites* | 5.8 | 3.8 | 116 | 568 | 7 | 1fok |
| 6 | Sporulation specific protein 16 from *Saccharomyces cerevisiae* | 5.5 | 3.5 | 86 | 197 | 9 | 6bzf |
| 5 | Zinc-dependent Dnase from *Escherichia coli* EC869 | 5.5 | 2.9 | 95 | 213 | 5 | 4g6u |
| 7 | EndoMS endonuclease from *Thermococcus kodakarensis* (strain ATCC BAA-918) | 5.4 | 3.4 | 91 | 239 | 13 | 5gke |
| 8 | Hypothetical protein AF1548 from *Archaeoglobus fulgidus* | 5.4 | 3.1 | 104 | 184 | 6 | 1y88 |
| 9 | Glycosyl transferase family 8 from *Anaerococcus prevotii* | 5.3 | 4.8 | 101 | 234 | 8 | 3tzt |
| 10 | Protein VC1899 from *Vibrio cholerae* | 5.2 | 3.3 | 106 | 380 | 7 | 1xmx |
| 11 | Cas4 nuclease SSO0001 from *Sulfolobus solfataricus* | 5.2 | 9.9 | 84 | 206 | 6 | 4ic1 |
| 12 | R.BspD6I-S subunit type IIS restriction endonuclease from *Bacillus sp.* D6 | 4.9 | 3.9 | 114 | 186 | 11 | 2p14 |
| 13 | Restriction endonuclease SdaI from *Streptomyces diastaticus* | 4.8 | 4 | 98 | 319 | 12 | 2ixs |
| 15 | Putative Aromatic Acid Decarboxylase from *P. aeruginosa* | 4.8 | 4.1 | 97 | 502 | 9 | 4ip2 |
| 16 | Putative decarboxylase FDC1 from *Aspergillus niger* (strain CBS513.88) | 4.8 | 4.4 | 95 | 499 | 6 | 4za4 |
| 14 | Restriction endonuclease BamHI from *Bacillus amyloliquefaciens* | 4.8 | 4.5 | 106 | 212 | 5 | 1esg |
| 17 | 3-octaprenyl-4-hydroxybenzoate decarboxylase (UbiD) from Escherichia coli | 4.7 | 4.1 | 91 | 474 | 9 | 2idb |
| 20 | Cu,Mo-CO Dehydrogenase (CODH) from *Oligotropha carboxidovorans* | 4.6 | 6 | 91 | 805 | 10 | 1n63 |
| 18 | Uncharacterized protein YqeQ from *Escherichia coli* | 4.6 | 4.9 | 99 | 180 | 9 | 3c0u |
| 21 | Human double-strand break repair protein Mre11A | 4.6 | 3.5 | 78 | 386 | 9 | 3t1i |
