# Supplementary figures and images for: Rhomboid intramembrane protease YqgP licenses bacterial membrane protein quality control as adaptor of FtsH AAA protease
Source: EMBO J. 2020 Jan 13;39(10):e102935. doi: 10.15252/embj.2019102935 (PMC7231995; doi:10.15252/embj.2019102935)

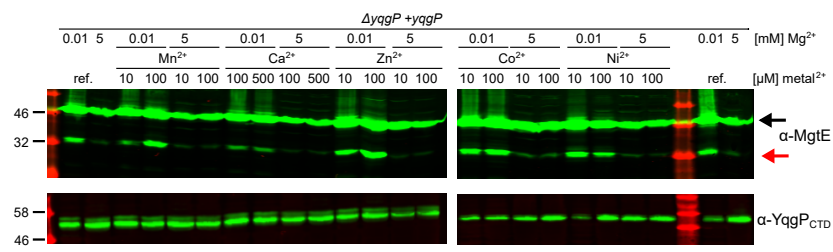

Supplement: Supplementary file 10 — Source Data for Figure 5 [file EMBJ-39-e102935-s009.pdf]
